# Case report: Chronic lymphocytic leukemia/small lymphocytic lymphoma and monomorphic epitheliotropic intestinal T-cell lymphoma: A composite lymphoma

**DOI:** 10.3389/pore.2022.1610653

**Published:** 2022-12-07

**Authors:** Bing Zhang, Yangyang Zhang, Quan Li, Qingjun Jiang, Wei Chu, Haifeng Gong, Ruyuan Li, Hong Ji

**Affiliations:** ^1^ Department of Urology, Qilu Hospital (Qingdao), Cheeloo College of Medicine, Shandong University, Qingdao, China; ^2^ Department of Pathology, Binzhou Medical University Hospital, Binzhou, China; ^3^ Department of Imaging, Binzhou Medical University Hospital, Binzhou, China; ^4^ Department of Imaging, Qilu Hospital (Qingdao), Cheeloo College of Medicine, Shandong University, Qingdao, China; ^5^ Department of Pathology, Qilu Hospital (Qingdao), Cheeloo College of Medicine, Shandong University, Qingdao, China; ^6^ Department of Gastroenterology, Qilu Hospital (Qingdao), Cheeloo College of Medicine, Shandong University, Qingdao, China

**Keywords:** case report, chronic lymphocytic leukemia, composite lymphoma, small lymphocytic lymphoma, monomorphic epitheliotropic intestinal T-cell lymphoma

## Abstract

**Background:** Composite lymphomas involving B-cell and T-cell lymphomas is very rare.

**Case presentation:** We reported a 63-year-old gentleman with composite chronic lymphocytic leukemia/small lymphocytic lymphoma (CLL/SLL) and monomorphic epitheliotropic intestinal T-cell lymphoma (MEITL). The patient was admitted to our hospital due to abdominal pain, and was diagnosed with CLL/SLL after bone marrow (BM) biopsy, BM aspiration, and flow cytometry. Two weeks later, he was diagnosed with MEITL based on pathological analysis after intestine excision. Next gene sequencing (NGS) findings identified two hotspot mutation sites (*STAT5B* and *DNMT3A*) closely related with the pathogenesis of CLL/SLL and MEILT. Additionally, *BCOR* mutation was only detected in the CLL/SLL area. The likely pathogenic mutations of CLL were *SETD2*, *NOTCH1*, *SF3B1*, and *PTPN11*, while the likely pathogenic mutations related with the MEILT were *TET2* and *ZRSR2*. Mutations of *GATA3*, *PLCG2*, and *FAT1* were identified in both CLL/SLL and MEITL areas, but the clinical significance was unknown. Finally, the patient died in the 12-month follow-up after surgery.

**Conclusion:** We report a rare case of composite CLL/SLL and MEITL that highlights the importance of careful inspection of hematologic neoplasms. We also present the results of NGS of different gene mutations in CLL and MEITL tissues.

## Introduction

Chronic lymphocytic leukemia/small lymphocytic lymphoma (CLL/SLL) is a typical indolent non-Hodgkin lymphoma (NHL) characterized by accumulation of malignant B cells in bone marrow (BM), peripheral blood and lymph nodes. Richter’s syndrome refers to high-grade NHL or Hodgkin lymphoma (HL) in patients with CLL/SLL. Approximately 2%–8% of patients with CLL/SLL would develop diffuse large B cell lymphoma (DLBCL), and less than 1% would present classic HL (1). In rare cases, CLL/SLL patients present T-cell malignancies with an incidence of approximately 1%, which is usually in the anaplastic large cell type or with a cytotoxic phenotype (2). Monomorphic epitheliotropic intestinal T-cell lymphoma (MEITL) is a rare primary intestinal T-cell lymphoma formerly known as type 2 enteropathy-associated T-cell lymphoma (EATL), resulting in very poor prognosis. In this study, we reported an extremely rare case presenting simultaneous CLL/SLL and MEITL.

## Case presentation

A 63-year-old gentleman was referred to our hospital due to needle-like pain in the upper abdomen for at least 15 days on November 18, 2020. The pain was transient and was relieved shortly, but he showed serious pain and vomiting occasionally. Gastroscopy showed reflux esophagitis and chronic atrophic gastritis. Doppler ultrasonography of the abdomen showed no abnormalities. Routine blood examination results were as follows: white blood cell, 17.9 × 10^9^/L (normal range: 4–10 × 10^9^/L); lymphocyte ratio, 62.5% (normal range: 20%–40%); lymphocyte, 11.0 × 10^9^/L (normal range: 1–3 × 10^9^/L); erythrocyte, 4.7 × 10^12^/L (normal range: 3.5–5.5 × 10^12^/L); hemoglobin, 87 g/L (normal range: 110–160 g/L); mean corpuscular volume (MCV), 67 fL (normal range: 82–92 fl); corpuscular hemoglobin (MCH), 19 pg (normal range: 27–31 pg); mean corpuscular hemoglobin concentration (MCHC), 284 g/L (normal range: 320–360 g/L); and ferritin, 8.21 ng/ml (normal range: ≥ 20 ng/ml). Upon physical examination, the patient showed bilateral inguinal lymphadenectasis. A smoking history was reported by himself with a duration of about 20 years (50 cigarettes per day). Nowadays, the patient does not smoke for 20 years. He had a history of drinking alcohol (about 150 g per day) for 40 years.

**FIGURE 1:**
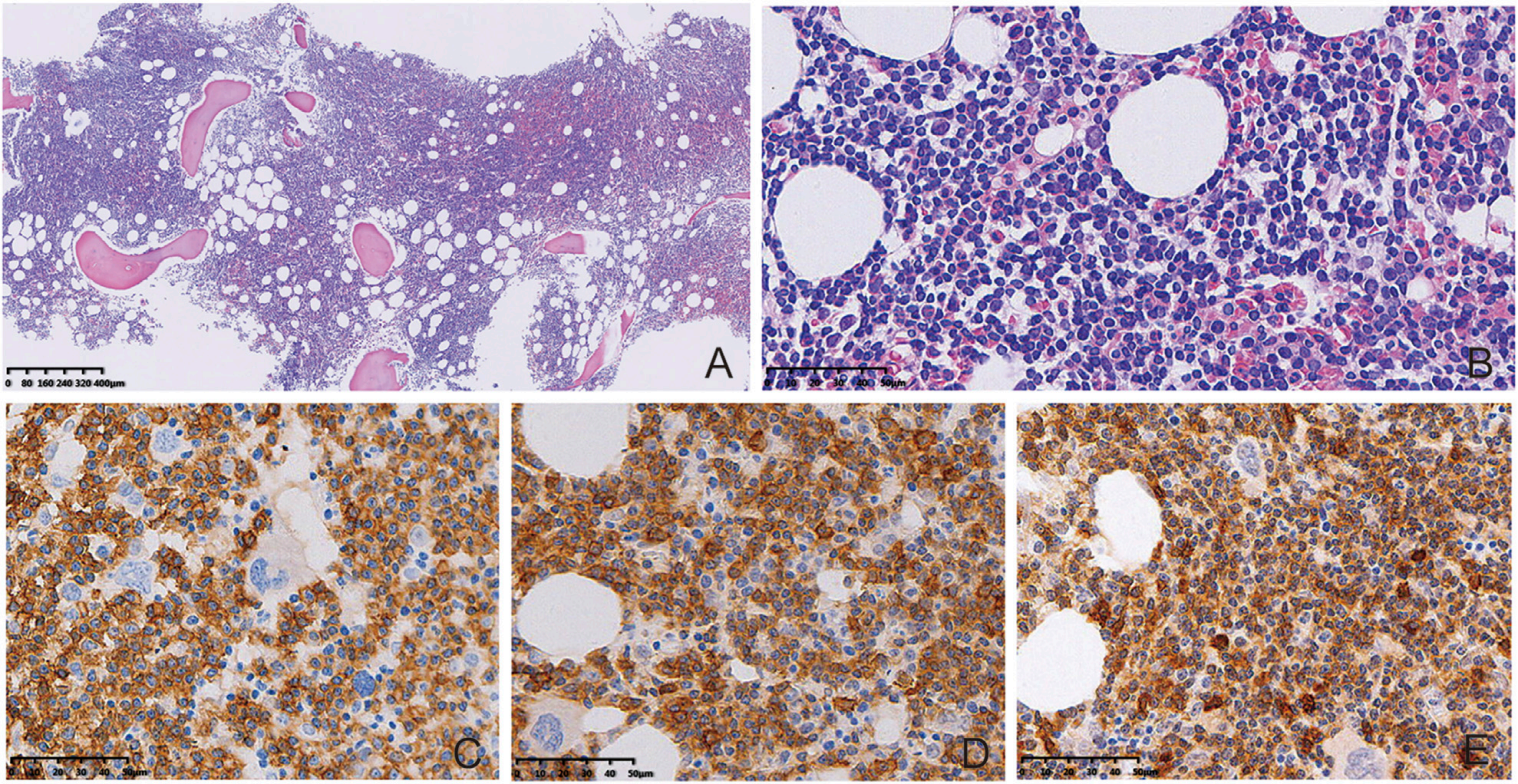
BM biopsy results. BM biopsy showed diffuse involvement by a small lymphocytic infiltration (**(A)**, HE, 40×). Presence of numerous small lymphocytes and scattered paraimmunoblasts in the infiltrated tissues (**(B)**, HE, 400×). The tumor cells were positive for CD20 (**(C)**, Enlivision, 400×), CD23 (**(D)**, Enlivision, 400×), and CD5 (**(E)**, Enlivision, 400×).

BM biopsy showed a markedly hypercellular marrow (85%), which was diffusely involved by a small lymphocytic infiltration (80%) (Figure 1A). The infiltration consisted of numerous small lymphocytes and scattered paraimmunoblasts (Figure 1B). The tumor cells were positive for CD20, CD5, and CD23, and were negative for CD3, CD10, TdT, and cyclin D1 (Figures 1C–E). For the aspirate smear, the majority (80%) were small lymphocytes with coarse chromatin. Peripheral blood smear showed scattered, small to medium sized lymphoma cells with irregular nuclear contour. Flow cytometry on BM aspirate demonstrated the presence of kappa restricted B cells, together with positive staining for CD19, CD20, CD22, CD5, and CD23. No *CCND1* gene break or *P53* gene deletion was identified by FISH, while no mutation was identified in *MYD 88* gene after Sanger sequencing.

**FIGURE 2:**
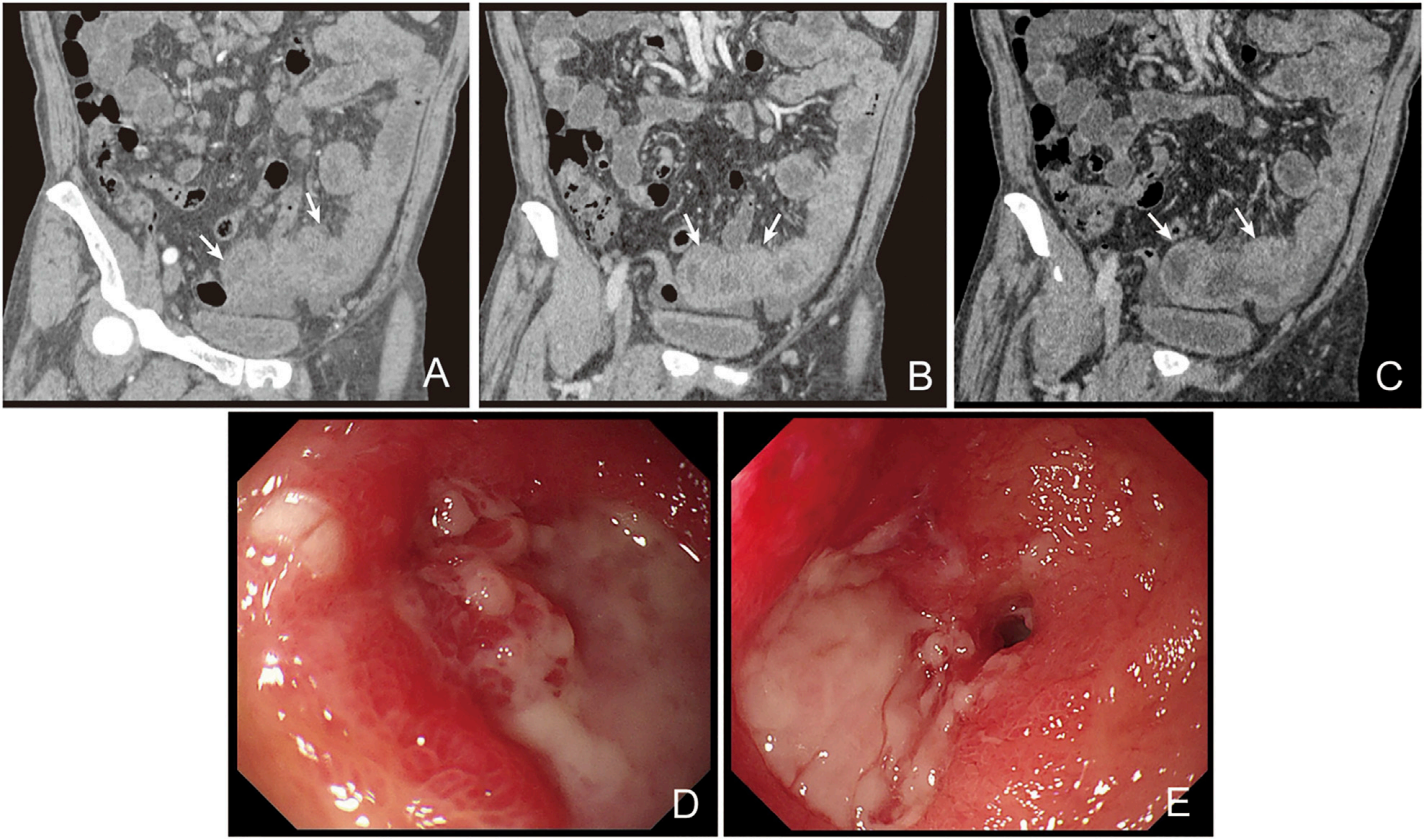
CT scan and enteroscope of the patient. CT enterograph of the small intestine showed even thickening in the wall of the small intestine (jejunum) in the pelvic cavity with multiple lymphadenopathy in enterocoelia and retroperitoneum (**(A)**, arterial phase; **(B)**, portal phase; **(C)**, lag phase). Enteroscopic examination showed multiple ulcers in the distal jejunum **(D)**. The ulcers were deeper with irregular margins and were covered with white pus moss. Enteroscopic examination showed stenosis in the jejunum **(E)**.

The patient was then diagnosed with CLL/SLL and microcytic hypochromic anemia simultaneously. Thoracic and abdominal CT showed lymphadenopathy in mediastinum, bilateral neck, axillary area, enterocoelia, retroperitoneal and pelvic cavity, as well as splenomegaly. CT scan indicated partial thickening in the small intestine. Thus, low-stage CLL/SLL was considered, and the patient merely received iron supplement for the treatment. However, the patient still reported persistent needle-like pain in the upper abdomen after treatment. CT enterography showed thickening in the small intestinal wall (Figures 2A–C), together with multiple lymphadenopathies in the abdominal cavity and retroperitoneum. Colonoscopy indicated three polyps with a size of 0.8 cm, 0.7 cm, and 0.4 cm, respectively. Pathological analysis indicated tubular adenoma with low-grade dysplasia. Finally, the patient was discharged with attenuation in the abdominal pain without treatment.

**FIGURE 3:**
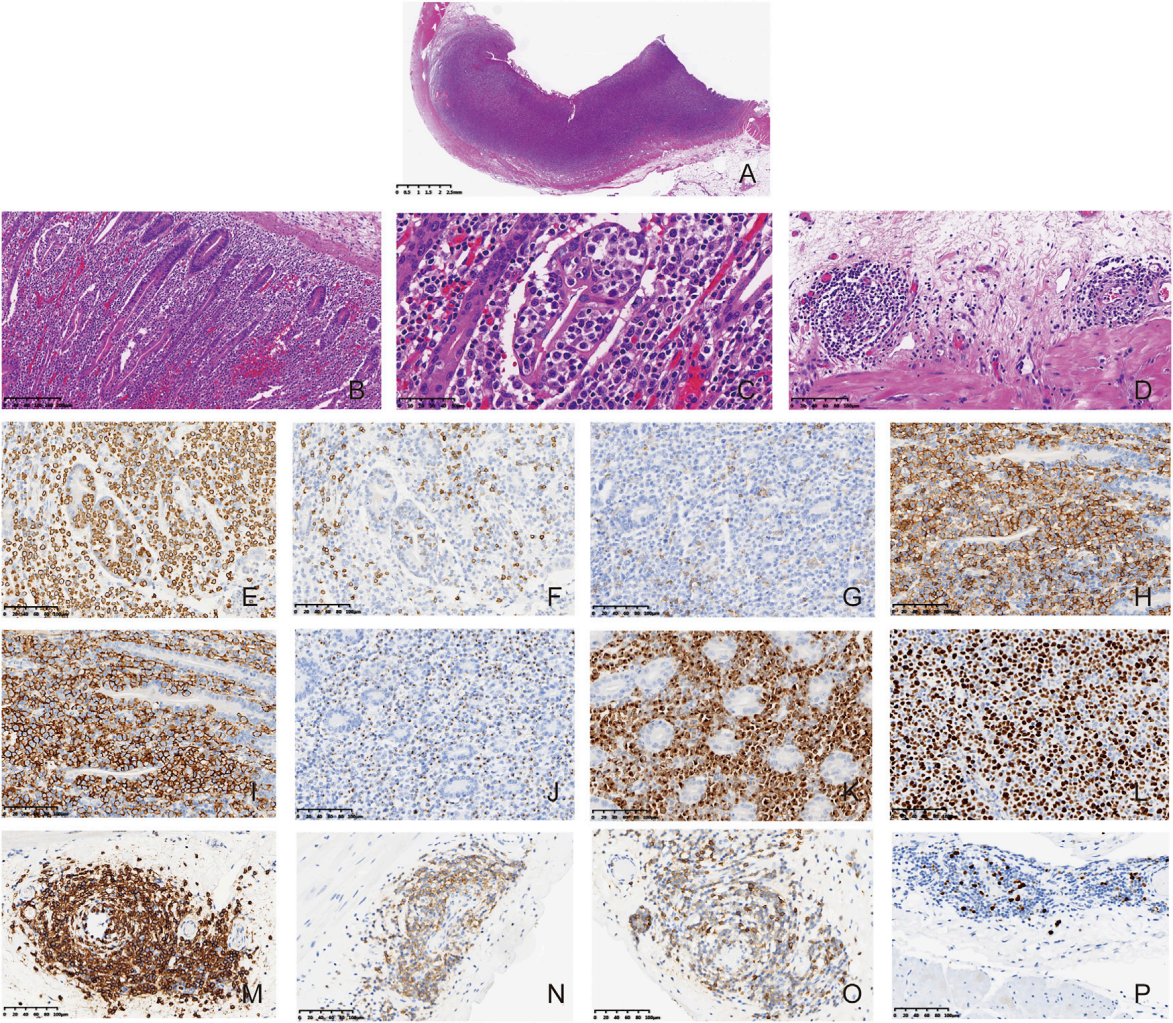
Composite CLL/SLL and MEITL. Microscopically, the intestinal wall was diffusely infiltrated by the neoplastic lymphocytes (**(A)**, HE, 8×). The villous architecture was distorted and the neoplastic cells showed prominent epitheliotropism (**(B,C)**, HE, 100×). The cells were generally medium in size with a generous rim of the pale cytoplasm. The nuclei were irregular, and most of them had finely dispersed chromatin and inconspicuous nucleoli. (**(C)**, HE, 400×) CLL/SLL tumor cells were found around the blood vessels in the intestinal wall at the periphery of the tumor (**(D)**, HE, 200×). The MEITL tumor cells in the mass were positive for CD3 (**(E)**, Enlivision, 200×), negative for CD5 **(F)**, Enlivision, 200×) and CD4 (**(G)**, Enlivision, 200×), positive for CD8 (**(H)**, Enlivision, 200×), CD56 (**(I)**, Enlivision, 200×), TIA-1 (**(J)**, Enlivision, 200×) and Granzyme B (**(K)**, Enlivision, 200×). The Ki-67 index of MEITL was in a range of approximately 70%–80% (**(L)**, Enlivision, 200×). The CLL/SLL tumor cells in the mass were positive for CD20 (**(M)**, Enlivision, 200×), CD23 (**(N)**, Enlivision, 200×), and CD5 (**(O)**, Enlivision, 200×). The Ki-67 index of CLL/SLL were about 5%–10% (**(P)**, Enlivision, 200×).

The patient showed recurrence of abdominal pain lasting for 2 weeks after discharge. Abdominal CT showed increase in the diameter and number of lymph nodes. Enteroscopic examination indicated multiple ulcers in the distal jejunum, which were deeper in site with an irregular margin covering them with white pus moss (Figures 2D,E). Based on the biopsy, inflammatory disease was considered by a general pathologist. Then he received laparoscopic exploration and partial enterectomy after endotracheal intubation and combined intravenous-inhalation anesthesia, which indicated multiple ulcers and narrowing in the small intestine. The intestinal wall was narrow and there was a mass at the position that was about 100 cm from the Treitz. Then a part of the intestine including the narrow lesion and the mass (2.5 cm × 1.0 cm × 0.5 cm) was excised. A narrowing site in the intestinal wall was observed at a distance of 2.5 cm from the mass. There was an ulcer in the lesion with a length and a diameter of 2.0 cm and 0.5 cm, respectively. Intestinal edema was noticed between the mass and the narrowed intestinal wall. The villous architecture was distorted in the mass and the whole layer was diffusely infiltrated by neoplastic lymphocytes. The villi were broadly expanded and infiltrated by the tumor cells, presenting prominent epitheliotropism in the peripheral intestine (Figures 3A–C). The cells were moderate in size with a generous rim of the pale cytoplasm. The nuclei were irregular, and most of them showed finely dispersed chromatin and inconspicuous nucleoli. Partial nuclei showed nuclear fold, prominent nucleoli, and dense chromatin. There was no inflammatory background or necrosis. Additionally, small lymphoid cells were seen around the blood vessels in the intestinal wall (Figure 3D).

The tumor cells were positive for CD3, CD7, CD8, CD43, CD56, TIA1, Granzyme B, and Bcl-2. In addition, the tumor cells were negative for CD20, CD79a, CD10, Bcl-6, MUM-1, CD23, cyclin D1, CD2, CD4, CD5, and CD30. The Ki-67 index was in a range of 70%–80% (Figures 3E–L). Tumor cells near the blood vessels were positive for CD20, CD79a, CD5, CD23, CD43, and Bcl-2. The Ki-67 index was in a range of 5%–10% (Figures 3M–P). The results were summarized in Table 1. At the focal edge area of the mass, there was mixed immunophenotype expression. The expression of EBV was negative in the tumor mass and the cells in the peripheral vessels. Finally, the patient was diagnosed with composite lymphoma with MEITL and CLL/SLL simultaneously. The peripheral blood and BM were not invaded by MEILT tumor cells based on the aspiration and the BM biopsy, which was confirmed by flow cytometry to the BM and peripheral blood.

**TABLE 1:** Clinical and pathological features of CLL/SLL and MEITL in the patient.

Antibody	Clone	MEITL	CLL	Antibody	Clone	MEITL	CLL
CD20	L26	−	+	CD2	AB75	−	−
CD79a	SP18	−	+	CD3	SP7	+	−
CD10	UMAB235	−	−	CD5	SP19	−	+
Bcl-2	SP66	+	+	CD7	EP132	+	−
Bcl-6	LN22	−	−	CD4	SP35	−	−
MUM-1	EP190	−	−	CD8	SP16	+	−
CD23	SP23	−	+	CD43	DF-T1	+	+
Cyclin D1	SA38-08	−	−	CD56	MX059	+	−
TIA-1	2G9A10F5	+	−	CD30	UMAB256	−	−
GranzymeB	EP230	+	−	Ki-67	30-9	70%–80%	5%–10%

There was clonal rearrangement for the IG detection using specific BIOMED-2 primers based on peripheral blood sample. Sanger sequence confirmed that it was IGHV3-33_06. The homologous degree was 100% compared with the germline sequence. PCR-based TCR assay revealed a small monoclonal T-cell population in a background of oligoclonal T-cells.

For the next-generation sequencing (NGS), B- and T-neoplastic cell content was estimated based on morphology, immunohistochemistry (IHC) and *IG/TCR* gene rearrangement. Two different paraffin blocks, with about 20% neoplastic cells in the HE sections, were selected for *IG* and *TCR* gene rearrangements and targeted sequencing. IHC indicated CLL/SLL harboring *IG* gene rearrangement, and MEILT harboring *TCR* gene rearrangement. Enrichment for areas of interest was scraped manually when comparing with the HE staining. High-throughput sequencing analysis was performed using haematopoietic and lymphoid specific panels covering the coding sequence (CDS) of 143 Hematological Disease genes through Illumina NextSeq 550 with a mean sequencing depth of 1000× (SINO-US Diagnostics Lab, Tianjin, China) (Supplementary Table S1). Data were analyzed using the bioinformatics pipeline in-house.

According to the NGS results, the pathogenic hotspot mutations of CLL/SLL and MEILT were *STAT5B* (c.1924A>C, p.N642H) and *DNMT3A* (c.2678G>A, p.W893*). Additionally, gene mutation on *BCOR* (c.1005dupC, p.S336Lfs*45) was only detected in the CLL/SLL area. The likely pathogenic mutations of CLL were *SETD2* (c.4688delG, p.G1563Afs*2), *NOTCH1* (c.7541_7542del, p.P2514Rfs*4), *SF3B1* (c.2902-2A>T), and *PTPN11* (c.1492C>T, p.R498W). The *TET2* (c.4094G>T, p.G1365V) and *ZRSR2* (c.1237A>G, p.K413E) mutations were likely pathogenic for the MEILT. Furthermore, mutations on *GATA3* (c.124C>T), *PLCG2* (c.1311T>G), and *FAT1* (c.7130C>T) were detected in both CLL/SLL and MEITL areas, which were classified as variants of uncertain clinical significance (UVS) (Table 2).

**TABLE 2:** The results of the NGS in CLL and MEITL areas.

Lymphoma	Mutated gene	Transcript ID	Mutated site	Nucleic change	Amino acid change	dbSNP	VAF (%)
**The pathogenic mutation**							
CLL	STAT5B	NM_012448	Exon 16	c.1924A>C	p.N642H	rs938448224	1.1
CLL	DNMT3A	NM_022552	Exon 23	c.2678G>A	p.W893*	rs750515748	2
CLL	BCOR	NM_017745	Exon 4	c.1005dupC	p.S336Lfs*45	—	23.1
MEITL	STAT5B	NM_012448	Exon 16	c.1924A>C	p.N642H	rs938448224	20.7
MEITL	DNMT3A	NM_022552	Exon 23	c.2678G>A	p.W893*	rs750515748	1.4
**The likely pathogenic mutation**							
CLL	SETD2	NM_014159	Exon 5	c.4688delG	p.G1563Afs*2	—	16.9
CLL	NOTCH1	NM_017617	Exon 4	c.7541_7542del	p.P2514Rfs*4	rs763016003	18
CLL	SF3B1	NM_012433	Intron 19	c.2902-2A>T	—	—	1
CLL	PTPN11	NM_002834	Exon 13	c.1492C>T	p.R498W	rs397507541	1
MEITL	TET2	NM_001127208	Exon 9	c.4094G>T	p.G1365V	—	8.4
MEITL	ZRSR2	NM_005089	Exon 11	c.1237A>G	p.K413E	RS766869777	1.6
**Variants of uncertain clinical significance**							
CLL	GATA3	NM_001002295	Exon 2	c.124C>T	p.P42S	rs774975933	41.3
CLL	PLCG2	NM_002661	Exon 14	c.1311T>G	p.S437R	—	47.2
CLL	FAT1	NM_005245	Exon 10	c.7130C>T	p.T2377M	rs201363601	45.9
MEITL	GATA3	NM_001002295	Exon 2	c.124C>T	p.P42S	rs774975933	49.5
MEITL	PLCG2	NM_002661	Exon 14	c.1311T>G	p.S437R	—	55.7
MEITL	FAT1	NM_005245	Exon 10	c.7130C>T	p.T2377M	rs201363601	42

For the follow-up, the patients did not receive additional treatment and passed away on January 6, 2022. This study was performed according to the convention of the Declaration of Helsinki, and the study protocols were approved by the Ethics Committee of Qilu Hospital (Qingdao). Written informed consent was obtained from the patient and his guardians.

## Discussion

Composite lymphoma is defined as coexistence of two distinct types of NHL or a rare combination of HL and NHL in a single organ or tissue (3), comprising 1%–4% of malignant lymphomas (4). Discordant lymphoma is defined as two distant sites involving two different lymphomas. In cases of sequential occurrence of two different lymphomas, it is known as a secondary lymphoma. Our patient was first diagnosed with CLL/SLL, and then was diagnosed with MEITL 1 month afterwards. The abdominal symptoms were presented at the first visit and the two NHLs were presented in the intestinal wall simultaneously. Therefore, the patient was diagnosed with composite lymphoma with CLL/SLL and MEITL.

In 1992, Strickler et al firstly reported 2 cases with nodal peripheral T cell lymphoma (PTCL) concurrent with CLL/SLL (5). Since then, 36 additional cases had been reported, among which 21 cases showed composite lesions involving both PTCL and CLL/SLL in the same biopsy (6). Among these 38 cases, 22 cases (57.9%) were qualified as PTCL or PTCL nos, including 12 with a cytotoxic phenotype, while 12 cases (31.6%) were ALCLs including 6 ALK-positive, 5 ALK-negative, and 1 ALK status unknown. The other 4 cases (10.5%) showed aggressive EBV-negative natural killer (NK)-cell leukemia (*n* = 1), EBV-positive nasal NK/T-cell lymphoma (n = 1), T-large granular lymphocyte leukemia (*n* = 1), and nodal lymphoma with a TFH cell phenotype (*n* = 1) (6). MEITL is a cytotoxic CD8^+^ T cell lymphoma, which accounts for the vast majority of primary intestinal T-cell lymphoma in Asia. Most MEITL cases show jejunal and ileal involvement. The neoplastic cells are featured by medium-sized round nuclei with a rim of pale cytoplasm, which usually infiltrate the intestinal epithelium. Our patient presented abdominal pain, and the medium-sized tumor cells infiltrated the intestinal wall with prominent epitheliotropism. Finally, the patient was confirmed with CLL and MEITL based on the immunophenotype analysis.

The patient was misdiagnosed as Inflammatory disease at first based on biopsy by a general pathologist. After reviewing the biopsy slides, we noticed atypical lymphoid cells in the background of necrosis and inflammatory exudation. Some tissues were crashed and the morphology was indistinguishable. Besides, there was no epitheliotropism in the biopsy. These may lead to misdiagnosis based on the biopsy. Furthermore, attention should be given to the morphology of the tissues, in order to avoid misdiagnosis.

In our patient, *STAT5B*, *DNMT3A*, *GATA3*, *PLCG2* and *FAT1* mutations were identified in both CLL/SLL and MEITL tumor cells. Activating mutations in *STAT5B* have been identified in a high proportion of MEITL cases (7-9). In a previous study, Diamantopoulos et al showed that the expression of STAT5B was correlated with the presence of EBV and LMP1, which was negatively correlated with the overall survival of the CLL patients (10). Somatic *DNMT3A* mutations were rarely identified in CLL and MEITL patients, however, low *DNMT3A* expression was associated with the pathogenesis of more aggressive diseases (11, 12). Trimech et al reported a small series of patients with composite lymphomas consisting of CLL/SLL and angioimmunoblastic T-cell lymphoma (AITL), in which the AITL comprised prominent clear cells with similar mutations consisting of TET2 or DNMT3A alterations (6). *DNMT3A* mutation was identified in both CLL/SLL and MEITL tissues in our case. As the expression of VAF was low, *DNMT3A* might be a manifestation of clonal hematopoiesis of indeterminate potential (CHIP) in case of the patient. In addition, *TET2* mutation was found in MEITL area rather than the CLL area, which was not frequent in MEITL patients (12). As the expression of VAF was 8.4%, we think TET2 mutation might be a passenger mutation or a manifestation of CHIP in these patients. Mutational analyses suggested that CLL progression on ibrutinib was closely related to the mutations of *BTK* and/or *PLCG2* genes (13). Monica et al reported that 10.3% of fludarabine refractoriness (FR)-CLL cases showed mutations of *FAT1* gene that encoded a cadherin-like protein involving in the negative regulation of Wnt signaling. On this basis, *FAT1* mutation may play important roles in the development of a high-risk phenotype (14). In our case, *PLCG2* and *FAT1* mutations were also detected in MEITL areas, and *GATA3* mutations were identified in CLL and MEITL. *GATA3*, *PLCG*, and *FAT* mutations showed similar VAFs in CLL and MEITL tissues, which indicated that the mutations in this patient were germline variants.

Mutations of *SETD2*, *NOTCH1*, *SF3B1*, *BCOR*, and *PTPN11* were identified in the CLL/SLL area of our patients. In a previous study, Parker et al. identified recurrent deletions of the SETD2 locus in 3% (8/261) of CLL patients, and they detected mutations of *SETD2* in an additional 3.8% of patients (23/602) based on NGS results (15). Their data highlighted *SETD2* aberration as a recurrent, early loss-of-function event in CLL pathobiology linked to aggressive diseases (15). However, the alterations in *SETD2* gene were more frequent in MEITL as recorded in 93% of Western European cases (16), and in 70% of cases from North America (17). In contrast, Chen et al. only reported two cases with *SETD2* mutation in Chinese MEITL patients (8). In our case, *SETD2* mutation was found in the CLL/SLL area, not the MEITL area. Mutations in *NOTCH1* and *SF3B1* were associated with a poor prognosis in CLL/SLL cases (1, 18). The *SF3B1* variant showed a very low allele burden and loss of function, which was not likely to be an oncologically relevant variant in this patient. Previously, Richter’s transformation had been considered to be associated with *NOTCH1* mutations (1, 19), and *BCOR* mutation was detected in 6.25% of CLL patients (20). This was the first report on *PTPN11* mutation in CLL and *ZRSR2* mutation in MEITL. However, the VAF of *PTPN11* and *ZRSR2* is very low, and further studies are required to investigate the unknown significance.

The following aspects may help to explain the pathology of composite lymphomas. Firstly, there was an underlying chronic immune dysregulation and T-cell stimulation in CLL patients. Certain factors secreted by CLL cells (e.g. inflammatory cytokines), may chronically stimulate normal lymphocytes, which then could lead to neoplastic transformation (21). In addition, studies on cellular immunity in patients with CLL/SLL reported reduced T-cell function with a paradoxical clonal expansion of CD8^+^ T cells and increased circulating CD8^+^ T-cell counts (22). This might explain the fact that the majority of T-cell lymphomas in CLL/SLL patients may have a cytotoxic phenotype, which expressed CD8 and/or cytotoxic granule proteins, in contrast to T-cell lymphomas in the general population (2, 6, 23-25). Secondly, composite lymphomas may be transformed from a stem cell with the possibility of developing into B-cell or T-cell lineages under different stimulations. Thirdly, the composited malignancies may not be directly related but may represent a shared genetic predisposition or step-by-step oncogenic potential. In cases with simultaneous diagnosis of CLL/SLL and T-cell lymphoma, it is not possible to determine which is primary. Nevertheless, in the present case, no specific treatment regimen was given to the CLL prior to diagnosis of MEILT. Additionally, both CLL and MEITL harbored *STAT5B*, *DNMT3A*, *GATA3*, *PLCG2*, and *FAT1* mutations. All these suggested that these two lymphomas may be derived from the same progenitor cells, while the different gene mutations might indicate that the progenitor cells received different environmental stimulation.

## Conclusion

In summary, we report a rare case of composite CLL/SLL and MEITL that highlights the importance of careful histopathologic inspection and immunophenotypic features of hematologic neoplasms. We also highlight the utilization of NGS in screening different gene mutations in CLL and MEITL tissues.

## Data Availability

The original contributions presented in the study are included in the article/Supplementary Material, further inquiries can be directed to the corresponding author.

## Abbreviations

AITL, angioimmunoblastic T-cell lymphoma; BM, bone marrow; BTK, Bruton tyrosine kinase; CHIP, clonal hematopoiesis of indeterminate potential; CLL/SLL, chronic lymphocytic leukemia/small lymphocytic lymphoma; EATL, enteropathy-associated T-cell lymphoma; FR, fludarabine refractoriness; HL, Hodgkin lymphoma; IHC, immunohistochemistry; MCH, corpuscular hemoglobin; MCHC, mean corpuscular hemoglobin concentration; MCV, mean corpuscular volume; MEITL, monomorphic epitheliotropic intestinal T-cell lymphoma; NGS, next-generation sequencing; NHL, non-Hodgkin lymphoma; PLCG2, phospholipase Cγ2; PTCL, peripheral T cell lymphoma.

## References

[1] Campo E Ghia P Montserrat E Harris N Müller-Hermelink H Stein H Chronic lymphocytic leukaemia/small lymphocytic lymphoma, who classification of tumours of haematopoietic and lymphoid tissues lyon. Lyon: IARC (2017). p. 216–21.

[2] Van Der Nest BM Leslie C Joske D Radeski D White R Cheah CY. Peripheral T-cell lymphoma arising in patients with chronic lymphocytic leukemia. Am J Clin Pathol (2019) 152:818–27. 10.1093/ajcp/aqz109 31433844

[3] National cancer institute sponsored study of classifications of non-hodgkin's lymphomas: summary and description of a working formulation for clinical usage. The non-hodgkin's lymphoma pathologic classification project. Cancer (1982) 49:2112–35. 10.1002/1097-0142(19820515)49:10<2112:aid-cncr2820491024>3.0.co;2-2 6896167

[4] Thirumala S Esposito M Fuchs A. An unusual variant of composite lymphoma: A short case report and review of the literature. Arch Pathol Lab Med (2000) 124:1376–8. 10.1043/0003-9985(2000)124<1376:AUVOCL>2.0.CO;2 10975943

[5] Strickler JG Amsden TW Kurtin PJ. Small B-cell lymphoid neoplasms with coexisting T-cell lymphomas. Am J Clin Pathol (1992) 98:424–9. 10.1093/ajcp/98.4.424 1329486

[6] Trimech M Letourneau A Missiaglia E De Prijck B Nagy-Hulliger M Somja J Angioimmunoblastic T-Cell lymphoma and chronic lymphocytic leukemia/small lymphocytic lymphoma: A novel form of composite lymphoma potentially mimicking richter syndrome. Am J Surg Pathol (2021) 45:773–86. 10.1097/pas.0000000000001646 33739791

[7] Tomita S Kikuti YY Carreras J Sakai R Takata K Yoshino T Monomorphic epitheliotropic intestinal T-Cell lymphoma in asia frequently shows SETD2 alterations. Cancers (Basel) (2020) 12:E3539. 10.3390/cancers12123539 PMC7759862 33260897

[8] Chen C Gong Y Yang Y Xia Q Rao Q Shao Y Clinicopathological and molecular genomic features of monomorphic epitheliotropic intestinal T-Cell lymphoma in the chinese population: A Study of 20 cases. Diagn Pathol (2021) 16:114. 10.1186/s13000-021-01173-5 34895266 PMC8667391

[9] Kim M Lee E Zang DY Kim HJ Kim HY Han B Novel genes exhibiting DNA methylation alterations in korean patients with chronic lymphocytic leukaemia: A methyl-CpG-binding domain sequencing study. Sci Rep (2020) 10:1085. 10.1038/s41598-020-57919-6 31974418 PMC6978354

[10] Diamantopoulos PT Sofotasiou M Georgoussi Z Giannakopoulou N Papadopoulou V Galanopoulos A Prognostic significance of signal transducer and activator of transcription 5 and 5b expression in EPSTEIN-Barr virus-positive patients with chronic lymphocytic leukemia. Cancer Med (2016) 5:2240–8. 10.1002/cam4.804 27367207 PMC5055175

[11] Biran A Yin S Kretzmer H Ten Hacken E Parvin S Lucas F Activation of notch and myc Signaling via B-cell-restricted depletion of Dnmt3a generates a consistent murine model of chronic lymphocytic leukemia. Cancer Res (2021) 81:6117–30. 10.1158/0008-5472.Can-21-1273 34686499 PMC8678341

[12] Mutzbauer G Maurus K Buszello C Pischimarov J Roth S Rosenwald A SYK expression in monomorphic epitheliotropic intestinal T-Cell lymphoma. Mod Pathol (2018) 31:505–16. 10.1038/modpathol.2017.145 29052597

[13] Quinquenel A Fornecker LM Letestu R Ysebaert L Fleury C Lazarian G Prevalence of BTK and PLCG2 mutations in a real-life CLL cohort still on ibrutinib after 3 years: A FILO group study. Blood (2019) 134:641–4. 10.1182/blood.2019000854 31243043

[14] Messina M Del Giudice I Khiabanian H Rossi D Chiaretti S Rasi S Genetic lesions associated with chronic lymphocytic leukemia chemo-refractoriness. Blood (2014) 123:2378–88. 10.1182/blood-2013-10-534271 24550227 PMC3983613

[15] Parker H Rose-Zerilli MJ Larrayoz M Clifford R Edelmann J Blakemore S Genomic disruption of the histone methyltransferase SETD2 in chronic lymphocytic leukaemia. Leukemia (2016) 30:2179–86. 10.1038/leu.2016.134 27282254 PMC5023049

[16] Roberti A Dobay MP Bisig B Vallois D Boéchat C Lanitis E Type II enteropathy-associated T-cell lymphoma features a unique genomic profile with highly recurrent SETD2 alterations. Nat Commun (2016) 7:12602. 10.1038/ncomms12602 27600764 PMC5023950

[17] Moffitt AB Ondrejka SL McKinney M Rempel RE Goodlad JR Teh CH Enteropathy-associated T cell lymphoma subtypes are characterized by loss of function of SETD2. J Exp Med (2017) 214:1371–86. 10.1084/jem.20160894 28424246 PMC5413324

[18] Tausch E Schneider C Robrecht S Zhang C Dolnik A Bloehdorn J Prognostic and predictive impact of genetic markers in patients with CLL treated with obinutuzumab and venetoclax. Blood (2020) 135:2402–12. 10.1182/blood.2019004492 32206772

[19] Kohlhaas V Blakemore SJ Al-Maarri M Nickel N Pal M Roth A Active Akt signaling triggers CLL toward richter transformation via overactivation of Notch1. Blood (2021) 137:646–60. 10.1182/blood.2020005734 33538798

[20] Kim JA Hwang B Park SN Huh S Im K Choi S Genomic profile of chronic lymphocytic leukemia in Korea identified by targeted sequencing. PLoS One (2016) 11:e0167641. 10.1371/journal.pone.0167641 27959900 PMC5154520

[21] Riches JC Davies JK McClanahan F Fatah R Iqbal S Agrawal S T cells from CLL patients exhibit features of T-Cell exhaustion but retain capacity for cytokine production. Blood (2013) 121:1612–21. 10.1182/blood-2012-09-457531 23247726 PMC3587324

[22] Goolsby CL Kuchnio M Finn WG Peterson L. Expansions of clonal and oligoclonal T cells in B-cell chronic lymphocytic leukemia are primarily restricted to the CD3(+)CD8(+) T-cell population. Cytometry (2000) 42:188–95. 10.1002/1097-0320(20000615)42:3<188:aid-cyto5>3.0.co;2-q 10861692

[23] Martinez A Pittaluga S Villamor N Colomer D Rozman M Raffeld M Clonal T-cell populations and increased risk for cytotoxic T-cell lymphomas in B-CLL patients: clinicopathologic observations and molecular analysis. Am J Surg Pathol (2004) 28:849–58. 10.1097/00000478-200407000-00002 15223953

[24] Went P Agostinelli C Gallamini A Piccaluga PP Ascani S Sabattini E Marker expression in peripheral T-cell lymphoma: A proposed clinical-pathologic prognostic score. J Clin Oncol (2006) 24:2472–9. 10.1200/jco.2005.03.6327 16636342

[25] Kanavaros P Boulland ML Petit B Arnulf B Gaulard P. Expression of cytotoxic proteins in peripheral T-cell and natural killer-cell (NK) lymphomas: Association with extranodal site, NK or tgammadelta phenotype, anaplastic morphology and CD30 expression. Leuk Lymphoma (2000) 38:317–26. 10.3109/10428190009087022 10830738

