# Supplementary material for: Case report: Chronic lymphocytic leukemia/small lymphocytic lymphoma and monomorphic epitheliotropic intestinal T-cell lymphoma: A composite lymphoma
Source: Pathol Oncol Res. 2022 Dec 7;28:1610653. doi: 10.3389/pore.2022.1610653 (PMC9768801; doi:10.3389/pore.2022.1610653)
Supplement: Supplementary file 1 [file Table1.DOCX]

**Supplemental Table 1** 143 genes in the haematopoietic and lymphoid specific panels relevant to lymphohematopoietic disease

| Gene | Detected area | Gene | Detected area | Gene | Detected area | Gene | Detected area |
| --- | --- | --- | --- | --- | --- | --- | --- |
| ABL1 | CDS | CSNK1A1 | Exon2-4 | KLF2 | CDS | RUNX1 | CDS |
| ANKRD26 | Exon1/5’UTR | CUX1 | CDS | KMT2A | CDS | SETBP1 | CDS |
| ARID1A | CDS | CXCR4 | CDS | KMT2D | CDS | SETD2 | CDS |
| ASXL1 | CDS | DDX3X | CDS | KRAS | CDS | SF1 | CDS |
| ASXL2 | CDS | DDX41 | CDS | MAP2K1 | Exon2-3 | SF3B1 | CDS |
| ATG2B | CDS | DIS3 | CDS | MAPK1 | CDS | SH2B3 | CDS |
| ATM | CDS | DNM2 | Exon8/13/16/18/20 | MAX | CDS | SMC1A | CDS |
| B2M | CDS | DNMT3A | CDS | MED12 | CDS | SMC3 | CDS |
| BCL2 | Exon2 | DNMT3B | CDS | MEF2B | Exon2-3 | SPEN | Exon11 |
| BCL6 | 5’UTR | EED | CDS | MPL | CDS | SRP72 | CDS |
| BCOR | CDS | ERG1 | CDS | MYC | CDS | SRSF2 | CDS |
| BCROL1 | CDS | EP300 | CDS | MYD88 | CDS | STAG2 | CDS |
| BIRC3 | CDS | ETNK1 | CDS | NF1 | CDS | STAT3 | CDS |
| BRAF | CDS | ETV6 | CDS | NOTCH1 | CDS | STAT5B | Exon11/13-18 |
| BRINP3 | CDS | EZH2 | CDS | NOTCH2 | CDS | SUZ12 | CDS |
| BTK | Exon5/11/14-19 | FAM46C | CDS | NPM1 | CDS | TAL1 | Exon3 |
| CALR | CDS | FAT1 | CDS | NRAS | CDS | TCF3 | Exon6/15/17 |
| CARD11 | CDS | FBXW7 | CDS | NT5C2 | Exon9-16 | TERT | CDS |
| CASP8 | Exon10 | FGFR3 | CDS | PAX5 | CDS | TET2 | CDS |
| CBL | CDS | FLT3 | CDS+Intron14 | PDGFRB | Exon18 | TNFAIP3 | CDS |
| CCND1 | CDS | GATA1 | CDS | PHF6 | CDS | TNFRSF14 | Exon1-6 |
| CCND2 | Exon4-5 | GATA2 | CDS | PIGA | CDS | TP53 | CDS |
| CCND3 | CDS | GATA3 | CDS | PLCG1 | CDS | TPMT | CDS |
| CCR4 | CDS | GNA13 | Exon1-4 | PLCG2 | CDS | TRAF3 | CDS |
| CD28 | Exon4 | ID3 | CDS | PPM1D | CDS | U2AF1 | CDS |
| CD58 | Exonn2-3 | IDH1 | CDS | PRDM1 | CDS | USP7 | CDS |
| CD79B | CDS | IDH2 | CDS | PRKCB | CDS | WHSC1 | CDS |
| CDC25C | Exon8 | IKZF1 | CDS | PRPS2 | CDS | WT1 | CDS |
| CDKN1B | CDS | IL7R | Exon5-6 | PTEN | CDS | XPO1 | CDS |
| CDKN2A | CDS | IRF4 | CDS | PTPN11 | CDS | ZBTB7A | CDS |
| CEBPA | CDS | JAK1 | CDS | RAD21 | CDS | ZMYM3 | CDS |
| CNOT3 | Exon2-5 | JAK2 | CDS | RBBP6 | CDS | ZRSR2 | CDS |
| CREBBP | CDS | JAK3 | CDS | RELN | CDS | DHX15 | CDS |
| CRLF2 | Exon6 | KDM6A | CDS | RHOA | Exon2-5 | FOXO1 | Exon1-2 |
| CSF3R | CDS | KIT | CDS | RPL10 | Exon5 | PRPF8 | CDS |
| MSC | Exon1 | NFE2 | CDS | TYK2 | CDS |  |  |
